# Comment on “Ultrasonographic scores for ileal Crohn’s disease assessment: better, worse or the same as contrast‑enhanced ultrasound?”

**DOI:** 10.1186/s12876-023-02882-5

**Published:** 2023-08-05

**Authors:** Kim Nylund, Kerri Novak, Rune Wilkens

**Affiliations:** 1 National Center of Ultrasound in Gastroenterology, Haukeland University Hospital, Bergen, Norway; 2 Division of Gastroenterology and Hepatology, Department of Medicine, University of Calgary, Calgary, AB Canada; 3 Digestive Disease Center, Division of Medicine, Copenhagen University Hospital - Bispebjerg & Frederiksberg, Copenhagen, Denmark

## Abstract

We read with interest the study by Freitas et al. comparing contrast-enhanced ultrasound (CEUS) and parameters from a time-intensity curve (TIC) with the SUS-CD score and IBUS-SAS score in patients with Crohn’s disease (CD) undergoing gastrointestinal ultrasound (GIUS) and ileocolonoscopy. The aim was to compare the accuracy of CEUS and aforementioned scores in predicting terminal ileal inflammatory activity in patients with CD. In this retrospective study of 50 patients, inflammatory activity was defined as a segmental score of SES-CD ≥ 7 in the terminal ileum. The study found 30 patients with active endoscopic disease demonstrating no significant difference between the “inactive” and “active” SUS CD and IBUS-SAS scores. However, the CEUS peak enhancement derived from the TIC was shown to be significantly different. The authors conclude CEUS was superior for detecting inflammation in the terminal ileum, as opposed to ultrasound scores relying on bowel wall thickness and color Doppler.

We read with interest the study by Freitas et al. [1] comparing contrast-enhanced ultrasound (CEUS) and parameters from a time-intensity curve (TIC) with the SUS-CD score [2] and IBUS-SAS score [3] in patients with Crohn’s disease (CD) undergoing gastrointestinal ultrasound (GIUS) and ileocolonoscopy. The aim was to compare the accuracy of CEUS and aforementioned scores in predicting terminal ileal inflammatory activity in patients with CD. In this retrospective study of 50 patients, inflammatory activity was defined as a segmental score of SES-CD ≥ 7 in the terminal ileum. The study found 30 patients with active endoscopic disease demonstrating no significant difference between the “inactive” and “active” SUS CD and IBUS-SAS scores. However, the CEUS peak enhancement derived from the TIC was shown to be significantly different. The authors conclude CEUS was superior for detecting inflammation in the terminal ileum, as opposed to ultrasound scores relying on bowel wall thickness and color Doppler.

First, we posit the study aim of Freitas et al. cannot be compared to the studies by Sævik [2] and Novak [3], as the former endeavors to separate inflammation from the absence of inflammation in a dichotomous manner, not aiming to construct a score. However, we would like to address multiple problematic aspects of this study, and here we will focus on three: the study design, definition of inflammation, and reliability.

The study by Freitas et al. [1] has a single-center, retrospective design, including a highly selected population. Inclusion criteria limited patients examined to those having GIUS, CEUS, and endoscopy, within 1 month, with unknown indications. Intestinal ultrasound utilizing CEUS bowel wall perfusion measurements for detection of inflammation is only standard of care in select centers worldwide. Why were the examinations performed? Over half the patients had an SES-CD ≥ 7, thus moderate to severe disease [4]. This alone presents a substantial selection bias. Also, the endoscopist was not blinded to the ultrasound findings. Retrospective studies rarely allow for consistent, high-quality documentation allowing for later re-assessment. The IBUS-SAS score [3] and suggested standardized triple cine loop recording (one in longitudinal, one in cross-section, and one with color Doppler imaging) were published after all scans included in this study were performed. Finally, the main parameter in the study, peak enhancement, is not defined; it is unclear whether the fitted TIC or manually reading provides the exact value, nor is the unit provided. In comparison, the study by Sævik et al. was a prospective multicenter trial with complete blinding and a clearly defined patient population, including *the full activity range of* patients with Crohn’s disease undergoing endoscopy [2].

Second, an SES-CD ≥ 7 threshold for inflammation categorizes all patients with an SES-CD of 6 and less as *remission*. This classification, therefore, could include patients with very large ulcers (minimum SES-CD 3), clearly not remission, lacking face-validity. In clinical trials, the most commonly used cut-off for inflammation is SES-CD > 2 [5].

Finally, a significant known issue when using CEUS for measuring inflammation is reproducibility, ease of use, and lack of external applicability due to lack of standardization [6]. This paper presented no data on inter-rater reliability, which was a major focus when designing the SUS-CD and the IBUS-SAS [2, 3]. The individual parameters used in these scores show excellent to good interobserver reliability, and both scores have excellent inter-rater reliability. In a direct quote from the discussion Freitas et al. state: “We emphasize that an advantage of CEUS is the generation of TICs as an objective parameter of bowel enhancement, as opposed to subjective IUS parameters included in the studied scores.” This is a bold statement, and Freitas et al. need to show that CEUS is reproducible and reliable. According to Fig. 1 in the paper [1], there is an apparent mismatch between the observed intensities and the fitted TIC [7]. The curve fit is not provided in the paper. However, the fitted curve utilized for this study is a wash-in curve (Personal correspondence with the author). This could partly explain the poor curve fit as a wash-in curve is fitted to data that contains both wash-in and wash-out information. Several groups have used CEUS for detecting disease activity, and in some cases, they even show good interrater reliability [8, 9] or repeatability [10]. Unfortunately, these are all single-center trials using the same ultrasound scanner. The need for larger, prospective multicenter studies to validate the use of contrast cannot be understated before concluding superiority over grey-scale activity assessment. The feasibility and reliability of the ultrasound indices performed at the bedside, independent of machine vendor or software, is imperative to support the wider clinical use of GIUS.

**Fig. 1:**
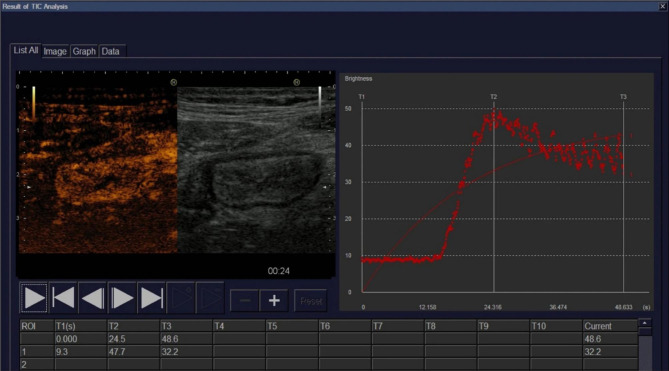
This shows Fig. 1 from the paper by Freitas et al. [1]. In the left panel the contrast image (left) is shown together with the B-mode image (right). In the right panel there are two red curves. The irregular curve are the actual intensity measurements over time. Every dot is a data point. The even curve is an approximation to these data points. The correspondence between the data points and the approximated wash-in curve seems to be poor

## Data Availability

Not applicable.

## References

[1] Freitas M, de Castro FD, Macedo Silva V, Arieira C, Curdia Goncalves T, Leite S (2022). Ultrasonographic scores for ileal Crohn’s disease assessment: better, worse or the same as contrast-enhanced ultrasound?. BMC Gastroenterol.

[2] Saevik F, Eriksen R, Eide GE, Gilja OH, Nylund K (2021). Development and validation of a simple ultrasound activity score for Crohn’s Disease. J Crohns Colitis.

[3] Novak KL, Nylund K, Maaser C, Petersen F, Kucharzik T, Lu C (2021). Expert Consensus on Optimal Acquisition and Development of the International Bowel Ultrasound Segmental Activity score [IBUS-SAS]: a reliability and inter-rater variability study on intestinal Ultrasonography in Crohn’s Disease. J Crohns Colitis.

[4] Koutroumpakis E, Katsanos KH (2016). Implementation of the simple endoscopic activity score in crohn’s disease. Saudi J Gastroenterol.

[5] Turner D, Ricciuto A, Lewis A, D’Amico F, Dhaliwal J, Griffiths AM (2021). STRIDE-II: an update on the selecting therapeutic targets in inflammatory bowel Disease (STRIDE) Initiative of the International Organization for the study of IBD (IOIBD): determining therapeutic goals for treat-to-target strategies in IBD. Gastroenterology.

[6] Serafin Z, Bialecki M, Bialecka A, Sconfienza LM, Klopocka M (2016). Contrast-enhanced Ultrasound for detection of Crohn’s Disease activity: systematic review and Meta-analysis. J Crohns Colitis.

[7] Wilkens R, Wilson A, Burns PN, Ghosh S, Wilson SR (2018). Persistent enhancement on contrast-enhanced Ultrasound studies of severe Crohn’s Disease: Stuck bubbles?. Ultrasound Med Biol.

[8] de Voogd F, Bots S, Gecse K, Gilja OH, D’Haens G, Nylund K. Intestinal ultrasound early on in treatment follow-up predicts endoscopic response to anti-TNFalpha treatment in Crohn’s Disease. J Crohns Colitis. 2022. 10.1093/ecco-jcc/jjac072 PMC9624292 35639823

[9] Ripolles T, Poza J, Suarez Ferrer C, Martinez-Perez MJ, de Martin-Algibez A (2021). Las Heras Paez B. evaluation of Crohn’s Disease activity: development of an Ultrasound score in a Multicenter Study. Inflamm Bowel Dis.

[10] Wilkens R, Peters DA, Nielsen AH, Hovgaard VP, Glerup H, Krogh K (2017). Dynamic contrast-enhanced magnetic resonance enterography and dynamic contrast-enhanced Ultrasonography in Crohn’s Disease: an observational comparison study. Ultrasound Int Open.

